# Real-Time Quantification of Gas Leaks Using a Snapshot Infrared Spectral Imager

**DOI:** 10.3390/s25020538

**Published:** 2025-01-17

**Authors:** Nathan Hagen

**Affiliations:** Department of Optical Engineering, Utsunomiya University, 7-2-1 Yoto, Utsunomiya 321-8585, Japan; nh@hagenlab.org

**Keywords:** gas detection, infrared imaging, leak rate, emissions quantification, spectral imaging, autonomous sensing

## Abstract

We describe the various steps of a gas imaging algorithm developed for detecting, identifying, and quantifying gas leaks using data from a snapshot infrared spectral imager. The spectral video stream delivered by the hardware allows the system to combine spatial, spectral, and temporal correlations into the gas detection algorithm, which significantly improves its measurement sensitivity in comparison to non-spectral video, and also in comparison to scanning spectral imaging. After describing the special calibration needs of the hardware, we show how to regularize the gas detection/identification for optimal performance, provide example SNR spectral images, and discuss the effects of humidity and absorption nonlinearity on detection and quantification.

## 1. Introduction

The continued improvement of gas sensors is an important goal for improving industrial safety and reducing fugitive gas emissions into the environment [1]. The most widely used sensor type for performing this is a “point sensor” that detects any gas passing across it or through it. However, the need for a point sensor to be inside a gas cloud in order to detect it means that industrial gas processing facilities must distribute hundreds of these point sensors across their sites in order to achieve sufficient coverage for explosion hazard safety warnings [2].

Another sensor type is a line sensor, which detects gas passing through a laser beam. Although line sensors can sense gas over a distance, and not just in their immediate area, their view is still limited to a single linear path. Filtered infrared cameras, on the other hand, can sense gas over an entire field of view, but unfortunately have limited ability to quantify their measurements since they require that the gas type be known a priori. In any situation where the gas type is not well known, such as when multiple gas types may be present, or in the presence of dust or steam, quantification is unreliable.

Attempting to overcome these limitations, researchers began developing imaging spectrometers for gas sensing [3,4,5]. While their combination of spatial and spectral information gives a platform for accurate quantification, their slow measurement speed and scanning artifacts have been problematic. Since about 2013, however, industrial gas sensing began to use snapshot infrared spectral cameras, which could deliver gas detection and quantification images in real time at video rate (15 Hz), and without scanning artifacts [6]. The improved detection performance of this approach allows for autonomous operation, in which the camera continuously monitors a field of view without direct human supervision [7].

Snapshot spectral imagers, unlike scanning imagers, can make extensive use of temporal correlations in the data in order to aid detection. They also typically have faster frame rates, allowing for imaging faster dynamics, and therefore smaller gas clouds. Spectral video combines more easily with standard visible-spectrum RGB video, allowing for easier integration of the extra visible-spectrum bands in the detection algorithm, particularly for removing potential false detections when changes in sunlight occur (e.g., due to changes in cloud cover), when dust or steam is visible, or when people or vehicles are moving within the camera’s field of view. Finally, for the case of the snapshot spectral imager considered here, the detection algorithm operates on a coarser spectral resolution than is typical for scanning systems—using spectral channels about 0.5 μm wide, instead of the higher 0.01 μm resolution typical of other instruments [8].

Although previous reports have presented the theory of gas detection and quantification for infrared spectral data [9,10,11,12], there are a number of algorithmic pitfalls that can present problems for accurate implementation. In the discussion below, we introduce the algorithmic data processing chain that has been implemented in a working snapshot infrared spectral camera, from raw camera images to gas detection to gas leak rate estimation. In particular, we focus on the algorithmic needs specific to such snapshot systems, such as how to take advantage of the temporal dimension in the data, and point out potential difficulties along the way.

The following list summarizes the steps in the data processing chain, numbered by the section below in which each step is discussed:

Section 2 (a): Use lookup tables derived from the static two-point calibration to convert the raw digital counts from each camera to blackbody temperature estimates (i.e., convert digital counts → °C).
Section 2 (b): Generate the dynamic calibration correction coefficients and correct the image temperature estimates, then crop and co-register each camera’s image.
Section 3: Use the known transmittance spectra of the filters on each camera to convert the temperature estimates to band-integrated radiance *M* (i.e., convert C∘→W m−2 sr−1).
Section 4: At each pixel in the image, estimate the background spectrum, and use it to estimate the absorption spectrum.
Section 5: For each gas in the instrument’s library, calculate the probability of gas presence given (i) the estimated absorption spectrum shape and magnitude, (ii) correlation among spatial and temporal neighbors, and (iii) the likelihood of any interferent gases or objects.
Section 7: Calculate the gas column density from the absorption value and the gas type.
Section 8: Using a video sequence of gas detection frames, estimate the volumetric gas flow and the leak source location.

## 2. Spectral Imaging Hardware and Calibration

The first few stages of the algorithm processing chain transform the raw data stream delivered by the imaging hardware into a series of calibrated spectral radiance datacubes. Although there are a number of ways to perform snapshot spectral imaging [13], one of the easiest methods is to construct an array of cameras and place a different narrowband filter in front of each. Other snapshot methods such as image slicing spectrometry or integral field spectroscopy use a series of lens elements, so that the light must pass a number of air–glass interfaces. In the thermal infrared; however, where glass refractive indices are high and anti-reflective coating materials limited, surface reflection losses become significant after transmitting through a series of 10 or more elements. As a result, minimizing the number of optical surfaces, such as with the camera array approach, helps to keep light throughput high.

The processing chain begins with a stream of 14-bit images from an array of 10 microbolometer cameras operating at their full 60 Hz frame rate. The system sums together four consecutive images from each camera, thus delivering 16-bit images at 15 Hz. This doubles image SNR while also reducing data load on the processing algorithms. Because the cameras are allowed to run freely, the data stream arriving at the frame aggregator is asynchronous, and can contain synchronization errors as large as 8 ms (i.e., half the frame rate). However, the process of summing four consecutive frames means that an 8 ms synchronization error for a frame period of 67 ms (frame rate of 15 Hz) corresponds to a 12% timing error. In practice, this level of timing error has not been problematic.

One major weakness of the camera array approach, particularly in the case of uncooled microbolometer cameras, is that maintaining radiometric calibration becomes difficult. The conventional two-point calibration approach fills each camera’s field of view, first with one shutter and then another. The two shutters are maintained at different temperatures, and are coated with well-characterized blackbody-simulating paints [14], allowing one to calibrate the infrared detector’s bias signal and gain from the two known input radiances. The calibration images in raw digital counts are then converted to effective blackbody temperature estimates (°C). However, microbolometers are notorious for calibration drift, so that immediately after the two-point calibration, one finds that the bias and gain values begin to wander unpredictably. While these are problematic enough for a single sensor, this behavior is a disaster for a multi-camera system if one needs radiometric data. Indeed, without this correction, a typical microbolometer camera array maintains sufficient radiometric accuracy for only a couple of seconds. Beyond this, detector drift produces apparent changes to the scene spectra, making gas measurement difficult.

In order to compensate for calibration drift, one approach is to use the temperature sensors embedded inside each camera and pre-calibrate the detector response for the entire range of temperatures one might expect to encounter [15]. While this is an attractive approach, the author had difficulty maintaining sufficient radiometric quality with it, though of course one cannot rule out implementation error. Instead, the approach taken for the gas imaging camera system is a “dynamic calibration”. This idea is based on the idea that if there is a source with known spectral radiance in the scene that is within the field of view of all of the cameras, then we can use each camera’s estimate of the source’s radiance to perform a calibration correction at the same time that the rest of the image is used for measurement (see Ref. [16] for details). This allows the system to apply calibration correction during every measurement frame. The trouble, of course, is that having a static source placed within the scene means that the camera must keep the reference source within view at all times. This would confine such cameras to use lenses with wide fields of view, which due to resolution limits would also constrict the range at which gas could be detected.

**Figure 1:**
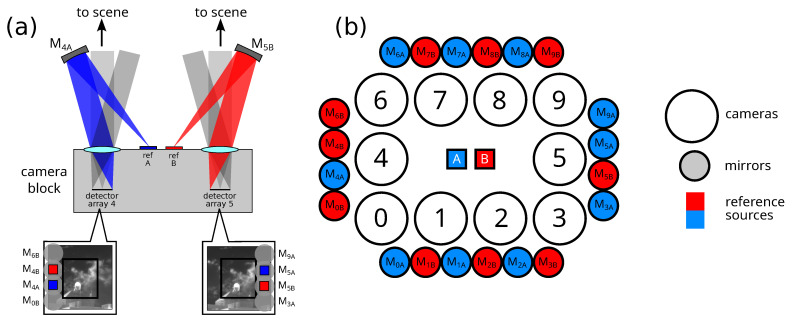
(**a**) A cross-sectional view of the camera array, showing only cameras #4 and #5. (**b**) An *en face* view of the camera array, together with the locations of the reference sources and the mirror array. Each mirror is labeled according to the camera (0 to 9) and reference source (A or B) that it is designed to image. (Image adapted from Ref. [12]).

The layout shown in Figure 1 illustrates one way around this limitation: we can place the reference source(s) at the center of the camera array, and then use relay optics to make sure that the reference sources (A and B) stay within every camera’s field of view. The dynamic calibration proceeds as follows. We obtain a corrected temperature estimate image $T^{\prime }\left(x,y\right)$ from an initial temperature estimate image T(x,y) from

(1) $$T^{\prime }\left(x,y\right)={\bar{T}}_{A}+\bar{\gamma }T\left(x,y\right)\;,$$

where $T_{A}\left(x,y\right)=W_{A}-\gamma \left(x,y\right)T_{A}\left(x,y\right)$ is the “dynamic offset”, and

$$\gamma \left(x,y\right)=\frac{W_{B}-W_{A}}{T_{B}\left(x,y\right)-T_{A}\left(x,y\right)}$$

is the “dynamic gain”. Here, $W_{A}$ & $W_{B}$ are the known temperatures of reference sources A and B, and $T_{A}\left(x,y\right)$ & $T_{B}\left(x,y\right)$ are the initial estimates of the reference source temperatures at pixel (x,y). While both the dynamic offset and gain calibration parameters $T_{A}\left(x,y\right)$ and γ(x,y) vary from pixel to pixel within the image of the reference source, we take their average values ${\bar{T}}_{A}$ and $\bar{\gamma }$ in order to apply them to correct the temperature estimates in the remainder of the image.

While this dynamic calibration approach does solve the calibration drift problem, it does require that we sacrifice a portion of each camera’s image for viewing the reference sources. In practice, this consumed about 25 pixels along the edge of the image. However, since the lower row of cameras (cameras #0–3 in Figure 1b) lose the bottom 25 pixels of their field of view, and the upper row of cameras (cameras #6–9 in Figure 1b) lose the top 25 pixels of their field of view, and likewise for the left and right edges, the actual loss amounts to about 50 pixels to both dimensions of the image. For a camera with a 320×240 pixel array, this amounts to 27% of the image.

For a filtered camera array, a complication to this dynamic calibration procedure is that each camera sees a different portion of the reference source spectrum. This is the reason for needing to use the optical temperature in order to match the cameras’ calibrations, rather than using their radiances directly. The reference source temperatures are values that every camera can agree on. Thus, in practice, each camera must have its own unique lookup table for converting raw digital counts to an estimated reference source temperature. If no calibration drift is present, then after this conversion every camera will estimate the same source temperature. As each camera’s calibration begins to drift, however, the individual cameras will need to be forced into agreement with one another—the dynamic calibration parameters are used to ensure that every camera’s source temperature estimate, and therefore the radiance estimate, agrees with every other camera.

Once we have the dynamically corrected temperature images $T^{\prime }\left(x,y\right)$ from each camera, the images are cropped to remove the regions blocked for calibration purposes, and each image is co-registered.

## 3. Spectral Channel Radiance Estimation

Microbolometer detector arrays are notorious for having severe noise, and the snapshot spectral imager used here is no exception. However, the multiaperture configuration of the system allows for a large increase in the light collection capacity of the instrument in comparison to scanning systems. A second method used to boost signal above the noise is to operate at low spectral resolution. Imaging 10 spectral channels across the 7.8–12.8 μm longwave infrared (LWIR) spectral range leads to a bandwidth of 0.5 μm per channel. Instruments such as scanning Fourier transform infrared spectrometers typically work with a much finer resolution than this. While the wider 0.5 μm channels allow for a large increase in signal, they have the drawback of nonlinear behavior in the presence of interference gases such as water vapor.

After obtaining the dynamically corrected blackbody temperature images $T^{\prime }\left(x,y\right)$, the next step is to convert these into radiance images M(x,y). Using the known transmission spectra of the filters $\tau_{w}\left(\lambda \right)$ on each camera, we can calculate the Planck blackbody radiance spectrum B(λ) and integrate over wavelength:

$$M_{w}\left(x,y\right)=\int_{\infty }B\left(\lambda ,T^{\prime }\left(x,y\right)\right)\;\epsilon \left(\lambda \right)\;\tau_{w}\left(\lambda \right)\;d\lambda \;,$$

where the subscript *w* corresponds to the filter index. For a system with 10 cameras, and therefore 10 filter functions, w∈{0,1,⋯,9}. The temperature $T^{\prime }$ corresponds to the blackbody temperature of the pixel, and ϵ represents the reference emissivity spectrum—in this case, the emissivity spectrum of the blackbody paint used for the calibration shutters and reference sources. From this point, we will make a distinction between *L* as a spectral radiance (units $W\;m^{-2}\;{\mathrm{sr}}^{-1}\;\mu m^{-1}$) and *M* as a total radiance value (units $W\;m^{-2}\;{\mathrm{sr}}^{-1}$). The exception is the blackbody radiance spectrum, for which we will use *B*.

**Figure 2:**
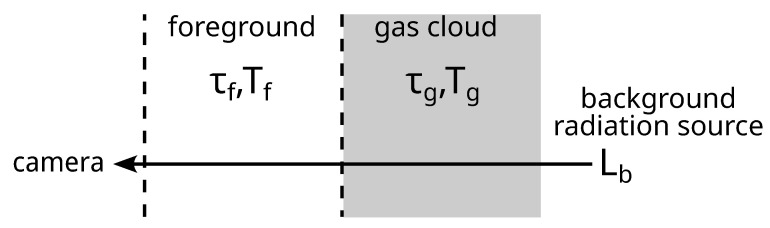
The measurement geometry for a single pixel in a gas cloud imager: the camera line of sight views the background infrared radiation through a gas cloud layer and a foreground atmospheric layer. Each layer has a spectral radiance *L*, transmission τ, and temperature *T*.

Gas imaging looks for changes in the radiance of a scene to indicate absorption or emission of gases passing between the camera and background [17]. This can be modeled as a three-layer radiative transfer system, as shown in Figure 2, where the background comprises some spectral radiance $L_{b}$ that must be estimated from measurements [18]. This background radiance traverses the gas cloud layer, and is attenuated/increased by absorption/emission of gases located there. The modified radiance then passes through an additional atmospheric layer to reach the camera. We use subscripts *f*, *g*, and *b* to represent the foreground, gas layer, and background of a physical quantity in each layer.

**Figure 3:**
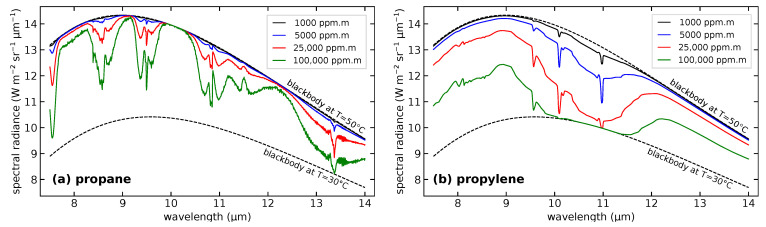
Radiance spectra for (**a**) propane and (**b**) propylene, for gas column densities from ξ=1000 ppm·m to 100,000 ppm·m, assuming a blackbody background at 50 °C and a gas temperature of 30 °C. The black dashed curves indicate blackbody spectra.

The absorption spectrum of a gas is defined by

(2) $$\alpha_{g}\left(\lambda \right)=1-\exp\left(-\sigma \left(\lambda \right)\;\int_{0}^{\ell }\rho \left(z\right)\;dz\right)\approx \sigma \left(\lambda \right)\;\rho \ell \;,$$

where *ℓ* is the path length through the gas cloud of concentration ρ, with absorption cross-section σ. The approximation used here assumes that the gas cloud is thin, so that we can ignore any nonlinear behavior in the exponential, i.e., α≪1. For a pixel in the scene observed by the camera, we can write the radiative transfer equation of a ray along the line of sight to give a spectral radiance of [9,10]

(3) $$\begin{array}{cc} L(\lambda ) & =\epsilon_{f}\left(\lambda \right)\;B\left(T_{f},\lambda \right)+\tau_{f}\left(\lambda \right)\;\epsilon_{g}\left(\lambda \right)\;B\left(T_{g},\lambda \right)+\tau_{f}\left(\lambda \right)\;\tau_{g}\left(\lambda \right)\;L_{b}\left(\lambda \right)\;d\lambda \\ \; & =\left(1-\tau_{f}\right)\;B_{w}\left(T_{f}\right)+\tau_{f}\left(1-\tau_{g}\right)\;B_{w}\left(T_{g}\right)+\tau_{f}\tau_{g}\;L_{b}\;. \end{array}$$

where the second step here uses Kirchhoff’s law for a thin gas, ϵ=α=1−τ. Figure 3 shows example L(λ) for propane and propylene gases.

## 4. Spectral Channel Absorption Estimation

Now that we have the estimated scene radiance images, we can analyze the spatial and spectral behavior of the video sequence to estimate what portions of the scene contain gas and what portions do not—a form of background-foreground separation algorithm [19,20,21].

Whereas the spectral radiance L(λ) is expressed in continuous form, the spectral imaging hardware measures the integrated radiance across the filter passband for each filter *w* in the system. To simplify the notation, we can write the integrals using angle bracket notation,

$${\langle S\rangle }_{w}=\int_{\infty }S\left(\lambda \right)\;\tau_{w}\left(\lambda \right)\;d\lambda \;,$$

for some spectrum S(λ) and weighting function τ. For some calculations, we will need a weighted average integral, for which we will use the double-angle-bracket notation,

$${\langle \;\langle S\rangle \;\rangle }_{w}=\frac{\int_{\infty }S\left(\lambda \right)\;\tau_{w}\left(\lambda \right)\;d\lambda }{\int_{\infty }\tau_{w}\left(\lambda \right)\;d\lambda }.$$

For each camera *w*, the imaging system delivers the radiance $M^{1}$ of the current frame and, from our background–foreground separation algorithm, an estimate of the radiance without gas present, $M^{0}$,

(4) $$M_{w}^{0}={\langle \epsilon_{f}\;B\left(T_{f}\right)+\tau_{f}L_{b}\rangle }_{w}\;,$$

and taking $\epsilon_{g}=\alpha_{g}=\left(1-\tau_{g}\right)$, the current-frame radiance is

$$\begin{array}{cc} M_{w}^{1} & ={\langle \epsilon_{f}\;B\left(T_{f}\right)+\tau_{f}\epsilon_{g}B\left(T_{g}\right)+\tau_{f}\tau_{g}L_{b}\rangle }_{w}\\ \; & ={\langle \epsilon_{f}\;B\left(T_{f}\right)+\tau_{f}L_{b}+\tau_{f}\alpha_{g}B\left(T_{g}\right)-\tau_{f}\alpha_{g}L_{b}\rangle }_{w}\\ \; & ={\langle \epsilon_{f}\;B\left(T_{f}\right)+\tau_{f}L_{b}\rangle }_{w}+{\langle \tau_{f}\alpha_{g}B\left(T_{g}\right)-\tau_{f}\alpha_{g}L_{b}\rangle }_{w}\\ \; & =M_{w}^{0}+{\langle \tau_{f}\alpha_{g}B\left(T_{g}\right)-\tau_{f}\alpha_{g}L_{b}\rangle }_{w}\;. \end{array}$$

Taking the difference between $M_{w}^{1}$ and $M_{w}^{0}$ (the “radiance contrast”) gives

$$M_{w}^{1}-M_{w}^{0}={\langle \tau_{f}\alpha_{g}\;B\left(T_{g}\right)\rangle }_{w}-{\langle \tau_{f}\alpha_{g}L_{b}\rangle }_{w}\;,$$

which does not yet lead to something we can use to solve for $\alpha_{g}$. However, in the thin gas case, only a small amount of error occurs if we assume separability, and thus calculate the integral of the product as a product of integrals, leading to

$$M_{w}^{1}-M_{w}^{0}\approx {\langle \tau_{f}\;B\left(T_{g}\right)\rangle }_{w}{\langle \;\langle \alpha_{g}\rangle \;\rangle }_{w}-{\langle \tau_{f}L_{b}\rangle }_{w}{\langle \;\langle \alpha_{g}\rangle \;\rangle }_{w}\;,$$

so that

(5) $${\langle \;\langle \alpha_{g}\rangle \;\rangle }_{w}\approx \frac{M_{w}^{1}-M_{w}^{0}}{{\langle \tau_{f}\;B\left(T_{g}\right)\rangle }_{w}-{\langle \tau_{f}L_{b}\rangle }_{w}}\;.$$

From (4), we can solve for the $\tau_{f}$-weighted background radiance,

(6) $${\langle \tau_{f}L_{b}\rangle }_{w}=M_{w}^{0}-{\langle \epsilon_{f}\;B\left(T_{f}\right)\rangle }_{w}\;.$$

Moreover, since $T_{g}\approx T_{f}$, and ${\langle \epsilon_{f}\;B\left(T_{f}\right)\rangle }_{w}$ = ${\langle \left(1-\tau_{f}\right)\;B\left(T_{f}\right)\rangle }_{w}$ = ${\langle B(T_{f})\rangle }_{w}-{\langle \tau_{f}\;B\left(T_{f}\right)\rangle }_{w}$, the terms in (5) can be substituted to give

(7) $${\langle \;\langle \alpha_{g}\rangle \;\rangle }_{w}\left(x,y\right)\approx \frac{M_{w}\left(x,y\right)-M_{w}^{0}\left(x,y\right)}{{\langle B(T_{f})\rangle }_{w}-M_{w}^{0}\left(x,y\right)}\;.$$

While this estimate for (7) is as unbiased as our approximations allow, we find that the denominator can often be quite small, and thus prone to large noise amplification. As a result, when using ${\langle \;\langle \alpha_{g}\rangle \;\rangle }_{w}$ for quantification, we used the above unbiased form, but when using ${\langle \;\langle \alpha_{g}\rangle \;\rangle }_{w}$ for gas detection, we use a regularized form that contains a small bias. For this purpose, it is usually sufficient to replace the $M_{w}^{0}\left(x,y\right)$ image term in the denominator with its spatial average ${\bar{M}}_{w}^{0}$:

(8) $$\left[\mathrm{regularized}\right]\;{\langle \;\langle \alpha_{g}\rangle \;\rangle }_{w}\left(x,y\right)\approx \frac{M_{w}\left(x,y\right)-M_{w}^{0}\left(x,y\right)}{{\langle B(T_{f})\rangle }_{w}-{\bar{M}}_{w}^{0}}\;.$$

If the resulting denominator is still small enough to cause noise problems, we add or subtract a scalar value (depending on whether the denominator is positive or negative) to nudge it out of its problem range.

**Figure 4:**
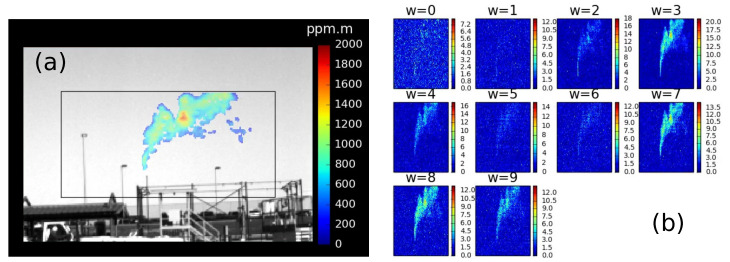
(**a**) A quantitative gas estimate overlaid (inside the black rectangle) on a monochrome visible image. (**b**) The SNR data for the same frame as (**a**), showing the SNR at each pixel for each of the 10 spectral channels of the system: each approximately 0.5μm wide from 7.8 to 12.8μm. The *w* label indicates the index of the spectral channel. (Note that the SNR image aspect ratios have been squeezed to half their original width in order to fit into the above layout).

An important quantity that we will need for the gas detection step is an estimate of the SNR at each pixel and camera. For this purpose, we apply an online updating algorithm [22] to the regularized absorption datacube ${\langle \;\langle \alpha_{g}\rangle \;\rangle }_{w}\left(x,y\right)$ that derives the SNR from continuously updating estimates of the mean absorption cube and the absorption variance cube. Figure 4 shows an example result.

## 5. Gas Detection Estimation

Once we have estimated the absorption datacube ${\langle \;\langle \alpha_{g}\rangle \;\rangle }_{w}\left(x,y\right)$ from the current frame, the next step is to determine whether we think that gas is present in each pixel of the scene, and how much of it. For the purpose of reducing computational load, it helps to approximate this as two independent probabilities: one probability operating on the shape of the spectrum (regardless of the magnitude), and a second probability operating on the magnitude, both conditional on the current estimate of the noise. We start with the probability estimate based on spectral shape.

Among the many spectral distance functions available, we use the SNR-weighted spectral cross-correlation. To do this, we first calculate the weighted variances and covariance of one spectrum a (the estimated absorption spectrum) and a second spectrum b (the reference gas spectrum),

$$\begin{array}{cc} \mathrm{cov}(a,b) & =\sum_{w}\omega_{w}\left(a_{w}-\bar{a}\right)\left(b_{w}-\bar{b}\right)\\ \mathrm{var}(a) & =\sum_{w}\omega_{w}{\left(a_{w}-\bar{a}\right)}^{2}\\ \mathrm{var}(b) & =\sum_{w}\omega_{w}{\left(b_{w}-\bar{b}\right)}^{2} \end{array}$$

for weights $\omega_{w}$, and for the average defined by $\bar{a}=\left(1/N_{w}\right)\sum_{w=1}^{N_{w}}a_{w}$, and likewise for $\bar{b}$. From these, we calculate the weighted cross-correlation as

(9) $$\mathrm{xcorr}\left(a,b\right)=\frac{\mathrm{cov}(a,b)}{\sqrt{\mathrm{var}(a)\;\cdot \;\mathrm{var}(b)}}\;.$$

**Figure 5:**
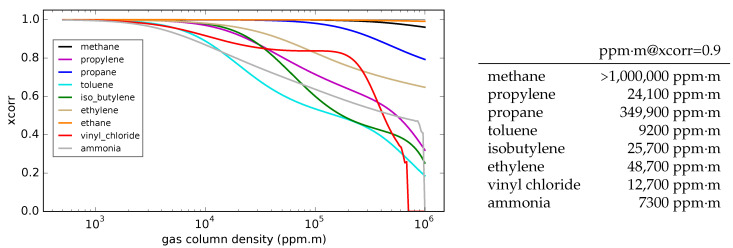
(**Left**) The reduction in cross-correlation as a result of gas absorption nonlinearity (i.e., approaching saturation). (**Right**) Gas column densities at which the cross-correlation of the gas spectra falls below 0.9 due to absorption nonlinearity.

If a given gas is present, and the signal has sufficiently good SNR, the absorption spectrum should provide a high correlation to the library gas cross-section spectrum [23,24]. However, at high gas column densities, the absorption spectrum shape changes due to nonlinearity of the absorption exponential α=1−exp(−σρℓ), as we see in Figure 3. Wavelengths with high absorption begin to saturate, so that the overall shape of the spectrum begins to deviate from the thin-gas shape. It is useful to know the column densities at which these effects begin to become significant. Figure 5 shows a series of cross-correlation values (with weights assumed to be 1 for all *w*) of different gases, with their corresponding library spectra, as a function of the gas column density. In each curve, it is assumed that the spectra are collected by 10 filters with 0.5 μm bandwidths across the 7.8–12.8 μm spectral range. The table in Figure 5 shows the gas column densities where the cross-correlation value falls to 0.9 due to absorption saturation. From these results, we can see that it is unlikely for the xcorr function to drop below any detection threshold for the case of methane. For the strongly absorbing gases, such as ammonia and propylene, however, the error can occur at gas column densities that one encounters in gas leaks. Note that the methane results of Figure 5 assume that the the measurement is unaffected by humidity—that the ambient air either has 0% humidity, or that the measured gas cloud is very close to the camera. Section 6 below discusses the interaction of methane and water vapor in more detail.

The absolute value of the weighted cross-correlation value can be considered as a rough estimate of the probability p(g) for the presence of gas *g* in the pixel,

$$p\left(g\right)\approx |\mathrm{xcorr}\left(\langle \;\langle \alpha_{g}\rangle \;\rangle ,\langle \;\langle \sigma_{g}\rangle \;\rangle \right)|\;.$$

In order to refine this estimate, we can make use of correlations that exist in our data. If gas is detected in a given pixel, then it is very likely to also be detected in its neighboring pixels, as long as background temperatures are similar between neighbors. This knowledge of the spatial correlations in the image plays a large role in improving detection performance. One can also take advantage of temporal correlations: gas detected at a pixel in one frame is likely to be detected in the same neighborhood in the following frame. These correlations can be used in a Bayesian updating procedure—to update the probability for gas presence, given the values of estimated gas absorption and of spectral cross-correlations in the pixel’s spatio-temporal neighborhood,

$$p^{\prime }\left(g\right)\propto p\left(\mathrm{data}|g\right)\;p\left(g\right)\;,$$

where we calculate the probability p(data|g) of the data, for the given gas *g*, from a Gaussian noise model, the absorption mean and variance spectra, and empirically derived correlation factors for spatial and temporal neighbors. Thus, the detection algorithm must make one pass through the image to estimate the initial probability p(g), and then a second pass using spatio-temporal correlations to update the estimate, giving $p^{\prime }\left(g\right)$.

When performing real-time processing on a high-volume data stream, however, this Bayesian update procedure can become too slow, and so faster approximate methods can be necessary. One approach is to use binary morphological operations, by thresholding the detection probability and then using erosion/dilation in the spatio-temporal directions to smooth out the detection region, but there is a significant loss in information (and therefore performance) by doing so. Grayscale morphological operations [25], on the other hand, are capable of more closely approximating the Bayesian spatio-temporal update step, generally providing a better tradeoff of performance for computational load.

So far, the detection process has focused entirely on positive detection—locating whether or not a given gas is present. For the purpose of reducing false detections, it is also important to detect the present of interferent gases and objects. One interferent that is ubiquitous in petrochemical processing facilities is steam. When a cloud of steam includes condensation water particles—a mix of dense vapor phase with liquid phase water—the shape of its absorption/emission spectrum is a family of curves that depend on the column density of vapor, and on the proportion of vapor to liquid phase, since the liquid form is strongly absorbing in the LWIR region. This greatly complicates the detection of mixed-phase steam from its thermal infrared spectral shape, but the broadband absorption/emission profile of liquid-phase water makes this form easy to detect. If water vapor (or in general any broadband absorption/emission poorly correlated with any library gas) is detected at a pixel then we update our detection probability downward accordingly.

When a cloud of steam includes liquid phase water particles, it can generally be seen in the visible-spectrum camera, so that the VIS camera image is also useful for the detection algorithm, as a way of preventing false detection. Almost all gases of interest are transparent in the visible spectrum, so that any significant changes in the scene that are not due to illumination change should be used to reduce the gas presence probability estimate,

$$p^{\prime }\left(g\right)\propto \left[1-p(\mathrm{data}|\mathrm{water})\right]\;p\left(g\right)\;.$$

A similar procedure also follows for using the visible camera to update the detection in the presence of dust clouds, which are another common feature of outdoor measurements among industrial equipment, especially when there are moving vehicles in the vicinity.

## 6. Effect of Water Absorption on Methane Detection

Methane is a gas of particular importance for industry, and yet has special difficulties when we try to detect it in the LWIR due to the partial overlap of its absorption spectrum with that of water vapor. Thus, the detected methane absorption signature varies with the path length in the foreground layer in Figure 2, the relative humidity, and the ambient air temperature. Using onboard sensors for the local air temperature, pressure, and relative humidity, one can easily calculate an estimate for the local concentration of water molecules. Assuming that the distribution of water vapor is homogeneous, the water vapor transmission spectrum is then

$$\tau_{\mathrm{water}}\left(\lambda \right)=\exp\left[-\sigma_{\mathrm{water}}\left(\lambda \right)\;\rho_{\mathrm{water}}\ell \right]\;,$$

for path length *ℓ*. The HITRAN database contains a detailed spectrum from which one can calculate the absorption cross-section $\sigma_{\mathrm{water}}\left(\lambda \right)$.

**Figure 6:**
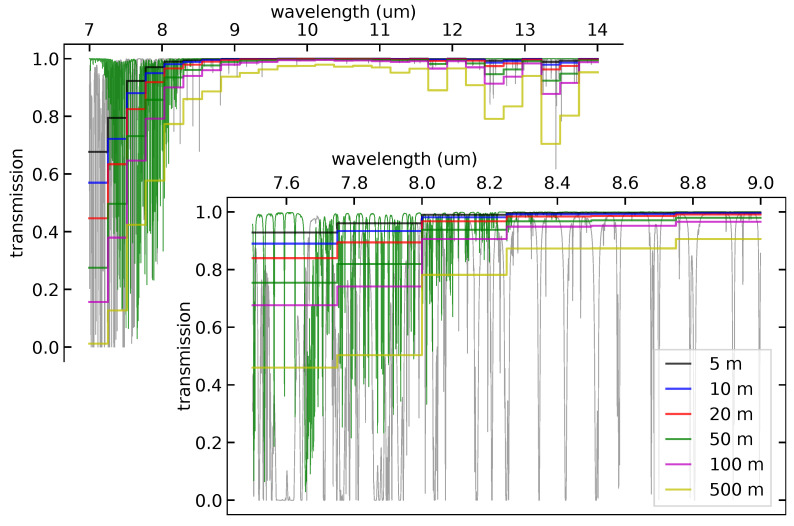
The water vapor transmission vs. path length through the air for 60% relative humidity and 25 °C air temperature. The thin gray curve shows the high-resolution water spectrum for a 10 m path length; the thin green curve shows the same for the methane spectrum, for a methane column density of 20,000 ppm·m. The stepped curves show the band-average transmission for water vapor averaged over 0.25μm wide bands, at a series of different path lengths from 5 m to 500 m.

In order to see how the water vapor and methane spectra overlap, Figure 6 shows high-resolution transmission spectra for water vapor and methane. The methane spectrum is calculated for a column density of 20,000 ppm·m, while the water vapor spectrum is calculated for 60% relative humidity, 25 °C air temperature, 1 atmosphere of pressure, and 10 m path length. The figure also shows the water vapor absorption averaged across 0.25 μm wide spectral bands, for a range of path lengths from 5 m to 500 m. We can see that as the path lengths increase, the band-averaged water vapor transmission increasingly obscures the methane signal, due to their overlapping absorption regions.

Because the methane absorption signature occurs at the beginning of the LWIR range, and over a relatively narrow range of wavelengths, its detection in the snapshot spectral imager primarily involves looking for an absorption signature where the first 0.5 μm wide transmission band w=0 shows a change, but all of the other bands w>0 show no change. Unfortunately, a cloud of water vapor (such as evaporating steam) produces a similar signature, making false detections of methane more probable than for other gases. As a result of this overlap, the detection thresholds for methane need to be set higher than would otherwise be necessary.

Indirectly, water vapor plays another role that interferes with methane detection. The presence of water vapor in the air means that the thermal contrast between the gas and the background spectrum will be reduced. Therefore, for view angles at which the background contains a long path length of water vapor, the background radiance at water vapor absorbing wavelengths effectively becomes the blackbody radiance of the air temperature. This reduces the radiance contrast signature $\Delta M_{w}=M_{w}^{1}-M_{w}^{0}$ to zero, if the gas under analysis is also at ambient temperature (as it usually is). This is why imaging methane in LWIR is generally performed with a geometry where a nearby physical object (such as a building or the ground) is used for the background, rather than the sky or some distant physical landscape. The shorter path length of a nearby background layer ensures that there can still be some radiance contrast with which methane can be measured.

## 7. Gas Column Density Estimation

At each pixel in the gas detection map, we know how to scale the absorption spectrum $\alpha_{g}\left(\lambda \right)$ at a pixel into gas column density ζ=ρℓ using the appropriate gas absorption cross-section $\sigma_{g}\left(\lambda \right)$. Several existing spectral libraries provide data on gases from which we can lookup the cross-section value $\sigma_{g}\left(\lambda \right)$ [24,26,27]. In the thin gas approximation, this conversion is simply

$${\langle \;\langle \alpha_{g}\rangle \;\rangle }_{w}={\langle \;\langle 1-\exp(-\zeta \;\sigma_{g})\rangle \;\rangle }_{w}\approx \zeta \;{\langle \;\langle \sigma_{g}\rangle \;\rangle }_{w}\;,$$

which we can invert to estimate the gas column density,

(10) $$\zeta_{g,w}={\langle \;\langle \alpha_{g}\rangle \;\rangle }_{w}\;/\;{\langle \;\langle \sigma_{g}\rangle \;\rangle }_{w}\;,$$

and, thus, averaging over all spectral channels,

(11) $$\zeta_{g}=\frac{1}{N_{w}}\sum_{w}{\langle \;\langle \alpha_{g}\rangle \;\rangle }_{w}\;/\;{\langle \;\langle \sigma_{g}\rangle \;\rangle }_{w}\;.$$

**Figure 7:**
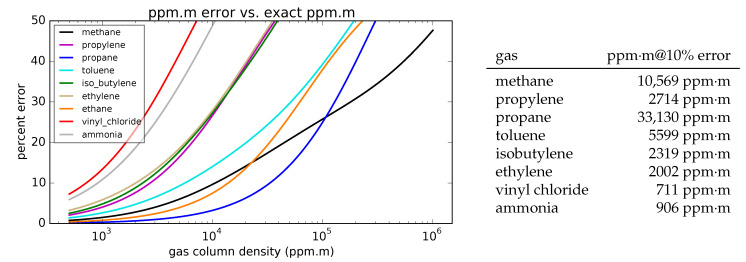
(**Left**) The percent error in the reconstruction gas quantity due to assuming a thin gas, as a function of the true gas column density. (**Right**) Gas column densities at which the quantification error reaches 10%.

Previous reports have discussed the accuracy that can be achieved with this quantification estimate with respect to measurement noise [28], and so here we focus instead on how the algorithm’s assumptions affect quantification accuracy. Several algorithm steps derived up to this point have assumed the gas to be thin (α≪1), it is easy to wonder how much of an effect this assumption has on the final result. Figure 7 shows the quantification error produced by using the thin gas assumption as a function of the true gas column density, assuming that they are being measured by a spectral imager having 0.5 μm wide spectral channels across the 7.8– 12.8 μm spectral range. Among the nine gases shown, spectra that have many sharp peaks and less broadband absorption will see more error due to the linearity assumption. The column density at which a given error occurs also depends on the magnitude of the absorption cross-section: since the propane absorption cross-section is low in comparison to the other gases, it experiences less error due to assuming linearity. (Once again, the methane results calculated here assume that either the ambient air has 0% humidity or the measured gas cloud is close to the camera.)

Regarding the impact of using the thin gas assumption, we can also add that for safety and leak detection and repair (LDAR) operations, low column densities are the most commonly encountered. Quantification error at high column densities is of lesser importance, since at these levels the user will likely already know that the gas cloud is a safety or a maintenance concern. For environmental monitoring purposes, we want both the thin and dense gas regimes to obtain accurate quantification. However, if we consider that current methods still struggle with achieving overall quantification accuracy within a factor of 2, the largest quantification errors are likely due to problems of viewing geometry, sufficient radiance contrast, presence of interferents such as water and dust, and quick dispersal of gas, rather than due to the smaller quantification errors that we see here due to the linearity assumption [29].

## 8. Gas Leak Rate Estimation

One of the triumphs of gas spectral imaging has been the successful achievement of autonomous gas leak rate estimation. Many early users of gas imaging cameras complained about the fact that the data are reported in units of column density (ppm·m) rather than the more intuitive units of concentration (ppm). However, column density units turn out to be critical for leak rate estimation.

The gas detection and gas column density estimation steps in the data processing chain deliver a video stream of quantified gas column density maps, which are overlaid onto a visible camera image to show the gas detection in real-time. With this video stream of quantitative gas maps, we can use optical flow based techniques to estimate the speed and direction of flow taken by each element within the gas cloud [30,31,32,33]. Tracking back through the optical flow estimate, we can also make a guess for the location of the leak source (from which all of the flow vectors originate), draw a border around the source, and calculate the volume of gas crossing this border. This is the gas leak rate, usually provided in units of L/m or g/s [7].

Without using a camera, estimating the flow rate of a gas leak is laborious: an engineer must seal the leak with a bag, pass the leaking gas through a tube and into a system that estimates the total flow and the concentration. This is a labor-intensive task that can take an experienced engineer over an hour of time. With a camera, a monitoring engineer need only setup the camera at a fixed point and start a detection algorithm running on the video stream. In good measurement situations, in which the leak source is within the field of view and the thermal contrast between the gas and the background is better than 10 °C, leak rate estimates obtained by averaging data over a period of several seconds have shown the ability to achieve errors of less than 20% [7].

## 9. Discussion and Conclusions

While most instruments used for gas imaging are either single narrowband filtered cameras or scanning imaging spectrometers [3,8,34], snapshot imaging spectrometers have begun to spread due to their improved light collection capacity, which can be used to help overcome the signal-to-noise problems associated with gas detection [28,35,36]. While gas imaging systems that use a single camera with a narrowband spectral filter are useful for a human user to interpret a scene, they have less utility for autonomous use. Obtaining quantified data with a single-filter system them requires that the user tell the camera what the gas is. Due to the presence of steam and dust, this is difficult to rely on for autonomous detection.

The algorithm summarized above provides a procedure for collecting infrared spectral images at a high rate sufficient to catch the dynamic behavior of many gas clouds, and to take advantage of spatio-temporal processing to improve detection performance. A recurring problem of all real-time algorithms operating on streaming spectral video, however, is a lack of computational resources to handle the high data flow rate. Most modern algorithms associated with detecting and tracking features in spectral images are focused on extracting the maximum amount of information from the data, rather than finding close approximations that deliver sufficient performance with limited resources. As a result, many of the algorithms used for this processing chain must be custom-built to the specific application.

A limitation in the discussion we have presented above is that it focuses entirely on single gases, rather than on mixtures of gases. In general, the literature has very little to say on the topic of estimation of mixtures, likely due to the difficulties involved. Because the gas detection must then account for a continuum of gas absorption spectra, the estimate becomes considerably less reliable, both in terms of noise behavior and of susceptibility to dynamic behavior in the background. It is the author’s considered opinion that, for mixture estimation, low-resolution passive multispectral imaging is likely inadequate for reliable estimation, so it is likely necessary to either use higher spectral resolution or to combine the current low spectral resolution imaging with active illumination high spectral resolution line-detection method to better extract mixture information.

As with all measurement approaches, camera-based approaches have specific strengths and weaknesses, and we have shown how the detection of methane in the LWIR comes with specific measurement needs for good data. That is, it requires a nearby rather than a distance object for background. We have also discussed the effects on both detection and quantification of the ubiquitous thin gas approximation, and why this is less of a problem than many may expect.

## Data Availability

The data presented in this paper may be obtained from the author upon request.
